# Should Informed Consent for Cancer Treatment Include a Discussion about Hospital Outcome Disparities?

**DOI:** 10.1371/journal.pmed.0050214

**Published:** 2008-10-21

**Authors:** Nadine Housri, Robert J Weil, David I Shalowitz, Leonidas G Koniaris

## Abstract

Background to the debate: Several studies have found disparities in the outcome of medical procedures across different hospitals—better outcomes have been associated with higher procedure volume. An Institute of Medicine workshop found such a “volume–outcome relationship” for two types of cancer surgery: resection of the pancreas and esophagus (http://www.iom.edu/?id=31508). This debate examines whether physicians have an ethical obligation to inform patients of hospital outcome disparities for these cancers.

## Abstract 2

Nadine Housri and colleagues examine whether physicians have an ethical obligation to inform patients of hospital outcome disparities for certain cancers.

## Nadine Housri and Leonidas Koniaris' Viewpoint: Physicians Have an Ethical Duty to Inform Patients of Hospital Outcome Disparities for Select Cancers

Thirty years ago, Luft and colleagues published the mortality rates for 12 surgical procedures of varying complexity in 1,498 hospitals, finding the mortality of open-heart surgery, vascular surgery, transurethral prostatectomy, and coronary bypass to be inversely related to hospital volume [1]. Since then, further studies have found an association between improved outcome and high hospital procedure volume [2–19] or teaching hospital status [20–23]. Such reports have led to calls for regionalization of care [12,24,25], and have served as the impetus leading consumer groups and policy makers to use hospital volume as a quality indicator and, in some cases, to direct patients to high-volume centers for select procedures [26,27] .

The results of outcomes studies, especially those for cancer, have generated significant interest among consumer groups and private organizations, some of which provide patients with hospital volume information on their Web sites [27]. In addition, a number of articles on volume–outcome studies have appeared in popular American newspapers and magazines [28–34]. These articles present a relatively simplistic view of volume–outcome data and may be subject to misinterpretation [35].

Since it is clear that patients are receiving information on hospital disparities in cancer treatment not only from their physicians but also from less informed sources, a crucial ethical question has arisen for the individual physician treating patients with cancer. Should primary care physicians, surgeons, and oncologists inform their patients of outcome disparities among hospitals? Should such information be disclosed as an element of informed consent? In response to one of the initial studies on volume–outcome relationships, Charles Culver and Bernard Gert argued in 1980 in favor of informing patients of volume–outcome disparities, where they exist [36]. However, almost 30 years later, it is questionable whether this information is disclosed during the informed consent process. Based on the principles of informed consent and the prevailing standards of disclosure, we argue that physicians have an ethical obligation to inform patients of hospital outcome disparities for select cancers.

### The Scope of Informed Consent in Cancer Care

Informed consent, which aims to protect the autonomous choice of the patient, is traditionally defined in terms of two components: (1) the disclosure of information on a procedure, leading to the patient's comprehension of this information; and (2) authorization by the patient to proceed with treatment [37]. Disclosure includes information on the nature of a procedure, potential risks and benefits, and alternative treatments. While patients are theoretically given adequate information to help them decide *whether or not* to be treated, they are given very little, if any, information to guide their decision on *where* to be treated. How often does the informed consent process include a discussion on the risks and benefits of treatment at a low-volume or nonteaching hospital or the possibility of an alternative treatment at a high-volume or academic center? Withholding outcome data where substantial differences in short- and long-term outcomes are observed potentially costs patients the ability to make an informed decision on where they should seek care.

### The Case for Informed Consent

Informed consent is often seen as a right of a patient and a duty or obligation of a physician [38]. Does a physician have an ethical obligation to share volume–outcome data when consenting a patient for treatment? And is it the patient's right to know this information when making a decision?

The necessity of disclosure can be assessed through two prevailing standards—the “professional practice” standard and the “reasonable person” standard. According to the “professional practice” standard, adequate disclosure is determined by the professional community's customary practices [37]. At the conclusion of the 2000 Institute of Medicine workshop examining evidence on the volume–outcome relationship, the National Cancer Policy Board determined that the volume–outcome relationship was strongest and most consistent for resections of the pancreas and esophagus [39]. While the volume–outcome relationship is gaining strength for a number of other cancers involving complex surgeries [5,6,10,11,13,22], the only cancer resections for which the Agency for Healthcare Research and Quality lists hospital volume in their *Inpatient Quality Indicators* are those of pancreatic and esophageal cancer. In addition, the Leapfrog coalition (http://www.leapfroggroup.org/), an alliance of large and small corporations that employs a total of over 20 million individuals, requires that employees who undergo one of five high-risk procedures must be cared for at high-volume centers. Two of the five procedures are pancreatic and esophageal cancer resections [40]. Therefore, we feel that the professional practice standard would, at the very least, support the disclosure of volume–outcome differences for patients undergoing pancreatic or esophageal cancer treatment.

Conversely, the “reasonable person” standard holds that the relevance of information is not based on the professional community's practices, but rather on the significance that a reasonable person would attach to it in making a decision [37]. What value would a reasonable woman with metastatic breast cancer place on the information that treatment at a teaching hospital is associated with a significantly higher chance of five- and ten-year survival? [22]. Would knowing that a local low-volume hospital may provide a man with rectal cancer with a significantly higher risk of a postoperative colostomy or even death cause him to seek out care at a high-volume center? [23] According to Finlayson's study on patient preferences for location of care, nearly half of surveyed patients stated that they would rather travel four hours to a distant center for a Whipple procedure than be treated at a local hospital if care at the distant center was associated with half the risk of operative mortality [41]. Interestingly, 45 out of 100 patients would not travel to a distant center if the risk was doubled at their local hospital, and almost one quarter of patients would rather face a six-times higher risk of operative mortality than travel for treatment. Thus while many patients may value outcome data in deciding where to have their surgeries, a similarly large number of patients will prefer local treatment, despite disparities in outcomes. The study makes no mention of the manner in which risks were framed when conveyed to the patient—“framing effects” during informed consent are known to influence patients' decisions [37]. The value that a reasonable person would place on outcome data in selecting location of care remains uncertain.

The reasonable person standard is one that can be very challenging for a physician to assess. In order to accurately evaluate what a reasonable person would find significant in making an informed decision, a physician must engage in discussion with the patient. Such discussion is also essential in allowing treatment to be a result of “shared decision-making” [38]. While informed consent and shared decision-making serve the same goal—to enhance a patient's control over his or her own medical care—they are two distinct entities. Informed consent is a physician's ethical and legal duty and mandates the two components outlined above: discussion of the procedure and explicit patient agreement or refusal. Shared decision-making, on the other hand, is a manner in which decisions may be made, involving an exchange of ideas between patient and doctor and collaboration on the decision itself [42]. We believe that for any cancer in which a volume–outcome relationship has been defined, discussion of this relationship should be part of both processes. Additionally, disclosure of outcomes research should be a required component of informed consent for those cases in which the volume–outcome relationship is significant and consistent, and is supported by the professional practice standard of disclosure.

### Consequences of Disclosure or Nondisclosure

Physicians may be hesitant in disclosing hospital outcome disparities for fear of migration of cancer patients from low-volume and nonteaching hospitals to high-volume and teaching hospitals. However, armed with knowledge of hospital-outcome data, patients will not necessarily choose to change hospitals—they may prefer to remain at a local hospital [31] or they may simply lack access to high-volume centers [43]. In addition, patients may not wish to travel to a high-volume center for cases in which treatment at a high-volume center confers only a small, albeit significant, advantage.

It is an ethical duty, and not fear of litigation, that should motivate physicians to provide full disclosure to their patients. Nevertheless, the legal implications of inadequate disclosure cannot be ignored. In the United States, the courts continue to differ in their definitions of adequate disclosure, and the states are split evenly between the two standards [44]. However, failures to inform patients of alternatives that may have otherwise yielded a better outcome have been met with legal action in recent years. In the case of *Johnson v. Kokemoor*, a neurosurgeon was successfully prosecuted for failure to accurately disclose risks of a basilar bifurcation aneurysm surgery performed by a surgeon at his level of experience. Furthermore, the Wisconsin Supreme Court decided that information on the availability of other centers and physicians better able to perform that procedure would have facilitated the plaintiff's awareness of “all of the viable alternatives” and her ability to make an informed choice [45]. A similar conclusion was reached in the Australian case of *Chappel v. Hart*, which sided with a patient plaintiff for failure of a treating physician to disclose the availability of a more experienced surgeon for a particular procedure [46]. “Loss of chance of a better outcome” is a recent development in Australian law in which negligence may be claimed without the need to prove causation. The loss of chance concept has been rejected by British courts. Nonetheless, more claims based on loss of chance are expected in Australia and may be successfully prosecuted unless rejected by the High Court [47].

### A Question of Trust and Honesty

Full disclosure of outcomes research, whether it is during the mandated process of informed consent or the recommended shared decision-making part of care, is essential to maintain trust and honesty in the physician–patient relationship. More importantly, it will protect the cancer patient's autonomy and sense of control, a value of paramount importance for a patient battling a potentially lethal disease.

## Robert J. Weil's Viewpoint: Such a Discussion Faces Logistic Hurdles and Risks Unintended Consequences

In their thoughtful and provocative viewpoint, Nadine Housri and Leonidas Koniaris propose that hospital outcome studies should be a necessary and obligatory component of the informed consent process ahead of offering surgery for cancer. Reviewing a number of the salient papers published over the past 30 years, they re-emphasize that, for some cancers, hospitals with higher volumes and/or teaching-training facilities have statistically lower rates of postoperative mortality and increased rates of survival (both indicators of surgical quality). In the field of oncological surgery, this association is true only of resections for esophageal and pancreatic cancer.

Housri and Koniaris' focus on both improving the delivery of health care and enhancing the shared decision-making process with patients is commendable. However, one must also consider carefully the broader effects of adding yet another requirement (and potential barrier) to the patient–physician relationship. Housri and Koniaris argue that surgeons have an “ethical obligation to inform patients of hospital outcome disparities for select cancers,” but their proposal faces a number of logistic hurdles. And, to quote Robert Merton, their proposal could have several “unanticipated consequences.” (In his seminal 1936 paper, “The Unanticipated Consequences of Purposive Social Action,” Merton outlined various types of unintended outcomes that may follow an organized, positive, and rational action [48].)

### Logistic Hurdles

There are a variety of hurdles to Housri and Koniaris' proposal. For example, it is unclear who exactly should disclose the hospital outcomes data to the patient. Does the burden of disclosure fall upon the surgeon or the hospital? It is also unclear what exactly should be disclosed and which types of hospitals should be compared. For example, should local teaching or high-volume hospitals be compared with local community hospitals, across states, populations, nations, or all three? Given advances in devices, medicine, surgical approaches, and technology, is it possible to compare hospitals or even recent time periods, especially when faced with disease courses that may extend over years? Similarly, it remains unclear how to compare the rates of surgical complications or successful outcomes in patients of variable surgical risk. And there are major hurdles in understanding, computing, and then conveying to the patient the difficult concept that statistics apply to populations but not to an individual.

### Unanticipated Consequences

One possible unintended consequence of mandating that physicians disclose hospital outcomes data is that hospitals or physicians may engage (overtly or subconsciously) in practices that help boost their hospital's rating. For example, in an effort to maintain good outcomes data, will community hospitals refer high-risk patients to the teaching hospital? Similarly, will surgeons do less radical or less morbid operations that may have little effect on cancer outcome but improve complication rates substantially? And what might happen in the event that a hospital is found to have poor surgical outcomes compared to other hospitals, and yet no specific causative factor is identified by careful audit, and the surgeon's *personal* outcomes are better than average? Should the surgeon switch hospitals?

What is most worrisome, when considering unintended consequences, is that most patients do not fully grasp the strengths and limitations of outcomes measures [41]. Indeed, Finlayson and colleagues found that many patients wish, for a variety of reasons and in spite of their physician's advice, to obtain care at a facility with a worse than average outcome [41].

### Weaknesses in Outcomes Studies

A universal desire among physicians is to enhance and promote a patient's welfare through improved outcomes. However, at present, outcomes studies remain incomplete for most cancers, frequently lag behind evolving standards of care for many tumors, and remain difficult to apply to the individual. For example, Koniaris and colleagues recently found that in the treatment of breast cancer, teaching hospitals were superior to community hospitals in terms of five-year mortality (*p* < 0.001) [22]. However, the disparity was only 3%–6% depending upon the type of community hospital, which may not represent a clinically meaningful difference, especially at the level of a single patient. Furthermore, as Pfister and colleagues noted [49], teaching hospitals tend to enroll more patients on clinical trials. In most of these trials, enrollment criteria require extensive evaluation to exclude metastatic disease, which may use technology available only at specialized centers (for example, PET imaging). In many of these cases, if patients do not qualify for the clinical trial due to previously unsuspected advanced or metastatic disease, they return to their community hospital and may be reported from that hospital, at their original stage, to the registry. This leads to a potential selection bias in favor of teaching hospitals in registries with respect to “initial stage” and overall outcome [49].

Given the potential variety of unpredictable outcomes for a given patient, the time may not yet be ripe to insist upon incorporation of outcomes data into the informed consent process. Efforts should be focused on processes that assist and stimulate physicians to improve patient care and outcomes, and on more incisive studies that determine the individual and organizational actions that lead to positive outcomes, and that can be applied universally. The value of Housri and Koniaris' viewpoint is that it should help stimulate our efforts to continuously seek improvements and refinements in the quality and performance of our care, so as to attain optimal results.

## David Shalowitz's Viewpoint: More Work Is Needed before Outcomes Data Are Routinely Used in Clinical Decision-Making

To what extent do volume–outcomes data matter in patients' decision-making? The National Cancer Policy Board of the Institute of Medicine concluded that “volume per se does not result in good outcomes in health care but is instead a proxy measure for other factors [potentially including] physician skill, experienced interdisciplinary teams, or well-organized care processes” [39]. The volume–outcome relationship is therefore less likely to be valuable to patients selecting a cancer treatment site than the actual outcomes data from potential treating institutions. Put another way, outcomes data from multiple institutions are likely to be important to a patient's selection of a treatment center, but the *causes* of differences in outcomes, whether case volume, teaching status, or otherwise, are far less likely to matter.

How, then, should outcomes data be communicated to patients? There are at least three possible strategies. The first is simply to ensure public access to hospital data, including outcomes data on different medical and surgical procedures, thus allowing the informed patient–consumer to select the best site for treatment.

However, despite the current emphasis on patient-centered medicine, we often prefer information critical to patient care to be communicated directly by a physician. A second strategy, therefore, would be for the physician to communicate outcomes data. When a patient has an established relationship with a primary care physician or specialist, outcomes data may be included in the process of determining what treatment course, if any, will be selected and where treatment will take place. For example, Housri and Koniaris note that while some patients may opt for treatment at the institution with the best outcomes, others may base their decision on proximity [41]. The shared decision-making interaction would likely best allow physicians to elicit patients' relevant preferences. However, this strategy would require physicians to obtain and interpret up-to-date outcomes data, which may be difficult and time-consuming.

A third option, as proposed by Housri and Koniaris, would be to incorporate outcomes data, where appropriate, into the consent form that the patient signs ahead of a surgical procedure—specifically in the “Risks” and “Alternatives to Treatment” sections of the form. On the one hand, surgeons may be best informed to discuss the relative risks and benefits of having a procedure performed at their institution versus another, and may be best placed to answer patients' questions. On the other hand, consent for a surgical procedure is solicited *after* the treating institution has been selected, and patients may still have many questions. Revisiting the selection of treatment site may consequently take time away from other important aspects of the consent process. Furthermore, including outcomes data at this point would make surgeons responsible for interpreting other institutions' outcomes data as well as their own, which may be neither possible nor prudent. Despite their best intentions, treating surgeons and institutions would be placed in the somewhat awkward position of determining whether to recommend a patient to another—perhaps competing—institution. This conflict of interest must be avoided whenever possible.

Housri and Koniaris' arguments do support a responsibility on the part of surgeons and hospitals to track their own complication rates and disclose these data accurately and understandably in the consent process for procedures. Competent adult patients may then decide whether the benefits and risks are compatible with pursuing the procedure. However, the ethical responsibility of hospitals to discuss other institutions' outcomes is still indeterminate. Selection of treatment site is, at this point, best done as part of a dynamic decision-making process in which all aspects of treatment can be evaluated in the context of the patient's preferences, including the importance of local treatment, differences in outcomes, existing relationships with surgeons, and other intangible considerations.

Importantly, Housri and Koniaris have also drawn attention to the content of the “Alternatives to Treatment” section of informed consent documents. This section has traditionally focused on alternative approaches to treatment rather than alternative treating physicians or institutions. However, careful examination of relevant ethical principles and legal precedents may prompt reconsideration of this standard. The role of outcomes data may be important to patients' treatment decision-making, but more work must be done to clarify: (1) patients' desire for outcomes data; (2) the proper arena(s) for communicating such data; and (3) the ethical/legal underpinnings of using outcomes data in treatment decision-making. Only then can discussion of outcomes data be mandated in selection of an institution for surgical care.
